# The interference effect of low-relevant animated elements on digital picturebook comprehension in preschoolers: An eye-movement study

**DOI:** 10.16910/jemr.17.4.1

**Published:** 2024-12-06

**Authors:** Nina Liu, Chen Chen, Yingying Liu, Shan Jiang, QianCheng Gao, Ruihan Wu

**Affiliations:** Tianjin Normal University, Tianjin, China; Institute of Psychology, Chinese Academy of Sciences, Beijing, China; No.26 Kindergarten, Hexi District, Tianjin, China; University College London, London, UK; Birkbeck, University of London, London, UK

**Keywords:** Digital Picture-Book, Animation, Eye Movements, Eye-tracking, Attention, Preschooler

## Abstract

Digital picture-book (DPB) with animated elements can enhance children’s engagement, but irrelevant animations may interfere with their comprehension. To determine the effect of the relevance of animated elements on preschoolers’ comprehension, an experimental study was conducted. Thirtythree preschoolers between the aged 4-5 years engaged with DPB in three conditions: high- and lowrelevant animations and a static control while listening to the story; their eye movements were recorded simultaneously. The study found that preschoolers had lower comprehension when exposed to low-relevant animation, but had comparable scores to the static condition with high-relevant animation. The results of eye-movement analysis showed that children who focused less on highrelevant or more on low-relevant elements had poorer comprehension. Those exposed to low-relevant animations looked less at high-relevant elements and more at low-relevant elements than those in the static and high-relevant conditions. These results suggested that low-relevant animations in DPB interfered with children’s comprehension by directing their visual attention away from crucial, highrelevant elements and more to less relevant elements. Therefore, designers creating DPBs, as well as parents and caregivers selecting DPBs for children, should consider the importance of the relevance of animated elements. And the corresponding mechanism of animation effect in DPB comprehension was discussed.

## Introduction

Picture books play an important role in cultivating and improving younger children's literacy development, especially when accompanied by a narrator (9; 16; 31; 41). With the rapid development of electronic technology, digital picture-book (DPB) emerge in virtue of digital interactive technology and multimedia presentation (2; 11). DPB contain rich multimedia resources, such as animated pictures, background music, sound effects and interactive function (37). An increasing body of research has been dedicated to investigating the effects of DPB on children, and the findings have shown that DPB can support learning even in the absence of parental mediation (e.g., 7; 12; 17; 23; 39).

A meta-analysis (42) has concluded that DPB have a small but significant positive effect on young children’s story comprehension and expressive vocabulary learning when compared to traditional picture book reading with a narrator (based on data from 2174 children in 43 studies). What’s more, it was showed that different multimedia features in DPB have different effects on children’s comprehension, for example, traditional multimedia elements, such as animation, music and sound effects, are considered to have promoting effects (10; 24; 38; 45; 46), while interactive elements, including hot spots and games, can distract children’s attention and have a negative impact (26; 28). However, in this meta-analysis, they divided multimedia features into two categories: traditional and interactive. Since each category contains many specific elements, it is difficult to pin down the true source of these effects. Therefore, to strengthen the effective utilization of multimedia resources in DPB, it is necessary to further investigate the mechanism of different types of multimedia resources in children’s DPB comprehension.

Animation is the most common multimedia presentation mode in DPB, which is importantly distinctive from the paper design of traditional PB. It has been found that the animated elements can arouse children’s interest in the corresponding picture content, so as to improve their understanding of the story (8; 40). Takacs and Bus (40) examined the effect of DPB containing only animated multimedia features on children’s comprehension. Thirty-nine 4-6 years old children read an animated and a static book while they listened to the story; and their eye movements were recorded at the same time. The results showed that, compared to static pictures, animation improved children’s comprehension significantly. What’s more, it is likely that animated pictures could improve story comprehension through directing children’s visual attention (19; 40; 48). Specifically, the eye-movement data showed that when the picture elements were animated and consistent with the story that children listened to, children looked the animated elements more than the static elements, even though these features did not provide more information. Moreover, children’s eye movements shifted less between different visual elements in the animated condition than that in the static condition, which is conducive to focusing more attention on important picture elements.

According to the dual coding theory proposed by Paivio (30), the human information processing system contains two channels, the auditory channel and the visual channel, which enable people to process both auditory and visual information in short-term memory. Children read picture books while listening to story text, they use visual information so that the pictures concretize the narration, thereby enabling dual-coding information processing (41). Animated elements enhance the integration of the picture content and the matching auditory story information by attracting children’s attention longer and more stable to important visual information. Specifically, the animated picture and the narration can contribute to the construction of a high quality mental representation of a story when animated pictures are strongly related to the narration. Thus, animated picture can improve children’s comprehension.

However, not all animated elements can improve children’s comprehension. In many DPB, animated technique is more used to “decorate” or increase the authenticity and richness, and animated elements are often chosen without regard to their relevance to the story topic. For instance, in a book named “*Clouds*”, clouds are the high-relevant elements as the story theme is to identify the weather through the changing shape of clouds. Compared with the elements of clouds, other elements like people and animals in the book are low- relevant to the theme. Bus and colleagues (8) argued that those low-relevant animated elements could distract children’s attention from the plot of the narrative content. When children focus more on the low-relevant picture elements, it would interfere with developing the mental representation of narrative (as it is not associated with the animated picture) and story comprehension will be compromised accordingly. Nevertheless, to the best of our knowledge, there is no direct evidence to support the interference effect of low-relevant animated elements.

Some potential evidence for this hypothesis was obtained in interactive DPB studies, in which they set low-relevant elements as the interactive elements. Trushell, Maitland and Burrell (43) compared children’s recall between interacting with an interactive DPB with hotspot animations (i.e. accessing the target animation with a mouse click) and merely reading it (i.e. the interactive function was turned off). The interacting group recalled significantly less story episodes than the reading group. It should be noticed that 75% of the animations were low-relevant to the story in the interacting group. A potential explanation is that too many low-relevant interactive hotspots can compromise children’s understanding of the story (49). Therefore, it is likely that the interference effect of this high interactive mode was caused by the excessive low-relevant elements in the interactive mode. Similarly, research into the effects of highly interactive DPB has found that DPB embedded with games increase inefficient interactions and interfere with children’s story comprehension (22).

In the current study, to investigate the relevance effect of animated elements on children’s story comprehension in multimedia learning, we directly manipulated the elements relevance. We included high-relevant animation condition (animated elements with high-relevance to the picture book theme), low-relevant animation condition (animated elements with low-relevance to the picture book theme), and a control condition (static elements). Furthermore, prior language skill, particularly vocabulary knowledge which is a crucial component for language development in Chinese (47), has been shown to influence children's DPB comprehension (21; 42), we also investigated how the relevance effect of animated elements on comprehension is affected by children's prior vocabulary knowledge. We expected that the animation of low-relevant elements would interfere with children’s comprehension, while the animation of high-relevant elements could promote their comprehension; and these might be caused by the change in visual attention distribution during DPB reading. Moreover, we expected that these effects would be influenced by individual differences in vocabulary knowledge.

Furthermore, the present study used eye-tracking technique to record children’s eye-movement behaviour while engaging with DPB. Since eye-tracking technique allows quick acquisition of objective and real-time analytics (5; 15), using this technique reveals children’s visual attention to varying animated elements. It could help us to further explore the mechanism underlying the animation effect in DPB comprehension. The popular science picture books were chosen as experimental materials. Given that the popular science pictures books are designed for conveying scientific knowledge (18) and have clean core elements which are closely related to the knowledge, it is easy for us to manipulate the elements relevance and to set the comprehension test.

## Methods

### Participants

Participants were 33 children in kindergarten aged 4.5 years on average (range from 4~5), and they were not familiar with the relevant science knowledge in the experimental materials. A sensitivity power analysis was conducted using G*Power (α=0.05, power=0.80), the results found that based on the existing sample size, the minimum effect size of the factor that we were able to detect was *f*=0.22, which was an effect between small and medium level (0.20<*f*<0.50) (13). Participants were native Chinese speakers and had normal or corrected-to-normal vision. None of them was diagnosed with any attentional or reading-related deficits.

### Material

(1) DPB materials. We selected three popular published science picture books: Clouds, Tales of Water and The Sunflower Story as reading materials. Given the popular science knowledge has not been taught in classroom teaching, the three books were evaluated by six teachers of our participants to ensure these materials are suitable for the reading ability of 4- and 5-year-olds.

To match the length across the three books, we unified the length of each book into eight pages (24 pages in total) without changing the plot. Moreover, to control the potential effect of text on evaluating children’s gaze on picture elements, we removed all the texts by Photoshop and recorded the text as the audio content of the picture book. All the recordings were made by a psychology graduate student from the faculty of psychology in Tianjin Normal University. Additionally, we controlled the audio content (e.g. removing unnecessary function words, modifying some expressions, etc.) to ensure it contained approximately 45 single-character words, resulting in about 12 seconds of audio per page. To confirm that these adjustments did not compromise story comprehension, fifteen undergraduate students were asked to carefully read each adapted book (with both picture and text presented simultaneously) and then rate the story’s completeness on a five-point scale (1 = very incomplete, 5 = very complete). The results showed that the integrity of all three books was high: *Weather Folklore*: *M* = 4.5 (*SD* = 0.64), *Tales of Water*: *M* = 4.3 (*SD* = 0.82); *The Sunflower Story*: *M* = 4.5 (*SD* = 0.64). This suggests that the length adjustment of the three books did not compromise story comprehension.

**Figure 1.**
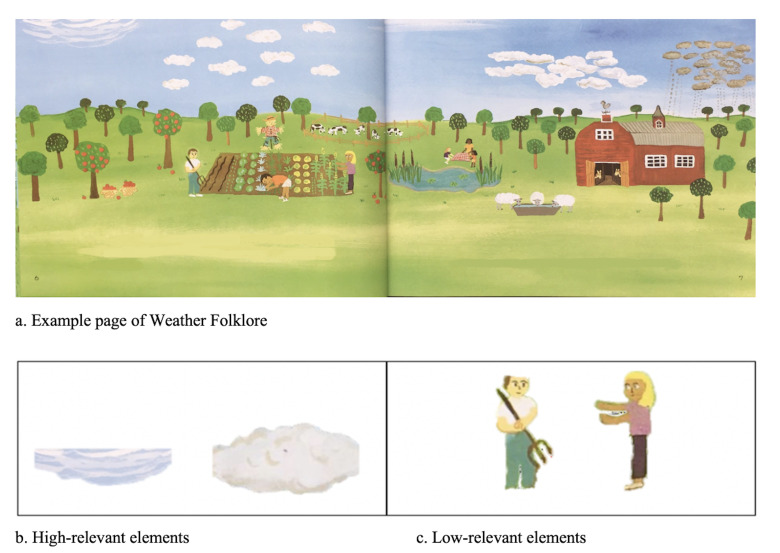
Target picture elements.

The target picture elements were selected by the following two steps. First, a psychology graduate student chose the high-relevant and low-relevant picture elements on each page of the book, using the core element of the theme as the criterion for judgment. For example, in *Clouds* (see Figure 1), the high-relevant elements were clouds in various shapes, while the low-relevant elements were people. Second, a different group of fifteen undergraduate students (who did not assess the completeness of the story) were asked to rate if they agree that selected elements were high-relevant or low-relevant, using a 7-point Likert scale (1= strongly disagree, 7 = strongly agree). Results showed that a high level of validity was achieved for the target elements of each book, *Weather Folklore: M* = 6.2 (*SD* = 0.85), *Tales of Water: M* = 6.3 (*SD* = 0.88); *The Sunflower Story: M* = 6.7 (*SD* = 0.72).

The selected picture elements were processed by using Flash and rendered together with the corresponding audio recording to generate the final video materials. In the animated high-relevant condition, only the high-relevant picture elements were moving throughout the frame; in the animated low-relevant condition, only the low-relevant elements were moving; in the static condition, the entire frame stays at rest.

**Figure 2.**
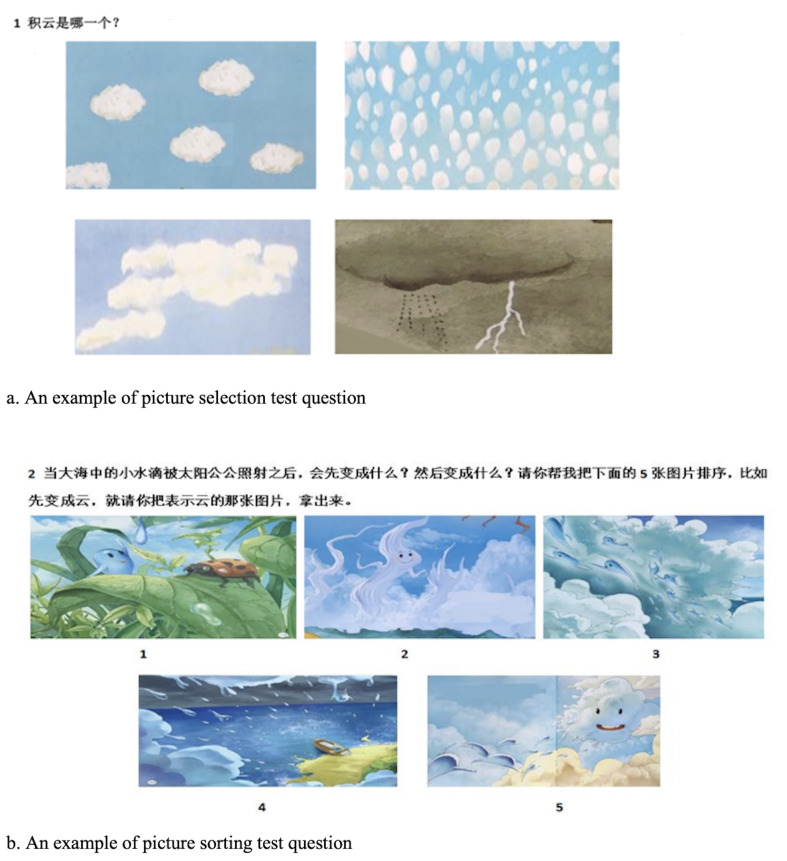
Examples of test questions. a. An example of picture selection test question. The question “Which one is cumulus?” contains four selections. b. An example of picture sorting test question. The question is “When the water drop in the sea is illuminated by the sun, what does it become first? What does it become next? Please help me to sort the following 5 pictures. For example, if it becomes clouds first, please take out the picture representing clouds.”

(2) Test materials. According to the content of each popular science picture book, a set of test questions was developed to investigate children’s acquisition of popular science knowledge after reading it. Questions consisted of pictures selection and pictures sorting. Picture selection questions adopted 2-point scoring (0 = wrong, 1 = correct). As showed in Figure 2A, the question “Which one is cumulus?” contains four selections, 1 point is for correct choice, 0 is for wrong choice. In picture sorting questions, participants received 0.5 points for each picture in the correct order, as showed in Figure 2B, “When the water drop in the sea is illuminated by the sun, what does it become first? What does it become next? Please help me to sort the following pictures. For example, if it becomes clouds first, please take out the picture representing clouds.” The total score for three books (*Clouds*, *Tales of Water* and *The Sunflower Story*) was 4, 5.5 and 6 points respectively.

(3) Test of vocabulary knowledge. We assessed children’s vocabulary knowledge by Peabody Picture Vocabulary Test-Revised ( PPVT-R; Dunn & Dunn, 1997; 36) . This test consists of 124 trials, in each of which 1 word and 4 pictures would be presented, and children were required to choose the picture that matches the word after hearing the word. One point was given per correct item. The test was terminated when the child made 6 mistakes across 8 consecutives items.

### Apparatus

A screen-based Tobii (Stockholm, Sweden) TX 300 eye-tracker system was used, and it with a sampling rate at 300Hz and data were recorded by Tobii Studio 3.1. Story videos were presented on a screen at 1920×1080 pixels. The distance between participants and the screen was approximately 64cm, and they were instructed to not move their head as much as possible during the assessment. The regular calibration type (5 points and the medium speed) was adopted, and the task started after the calibration reaching the standard. Calibration quality was evaluated by ensuring that error vectors were within a 1-degree range around each calibration point (approximately twice the diameter of the calibration point) based on empirical guideline. The "Verify" option was also used to double-check, ensuring that the participant's gaze overlapped with the calibration dots as required (see Tobii Studio User’s Manual for versions below 3.3 or earlier).

### Procedure

Participants were tested individually. Upon entering the testing room, each child's eye tracker was adjusted to ensure both eyes were detectable and children were comfortable, followed by the calibration procedure. After that, the instruction was presented (experimenter read it to the participants) as follows “Today we will read an interesting picture book together, please read it carefully, after reading the book you will be asked some questions. Are you ready? Let’s practice first!”

In practice section, an additional picture book was used as practice material to familiarize participants with the task. And after practice but before the formal task, the experimenter repeated the instruction again orally to make sure each child understood it correctly. In experiment section, each child read all three books, and each book was matched with one of the three conditions. To reduce children’s fatigue, the experiment was divided into three days with each child read one of the three books every day. We made the counterbalance across conditions and test days. Each day, the child began by familiarizing themselves with the instructions, completing eye-tracking calibration and practicing, followed by viewing one of the videos (DPB). This entire process took approximately 15 minutes. After the viewing session, the child moved on to the testing session, which lasted around 10 minutes. This study was approved by the Scientific Research Ethics Committee of the Institute of Psychology and Behavior at Tianjin Normal University, with the approval number (2019022801).

### Data Analysis

Data were analyzed by liner mixed-models (LMM) (1) using the lmer function from the lme4 package (Bates et al., 2011) within R (version 4.3.1, 33). For the dependent measure, a model was constructed with elements relevance, vocabulary knowledge (abbr. vocabulary, using continuous PPVT score centered on its mean) and the interaction of the two factors as fixed factors, using the contr.sdif (MASS) function (44). Participants and items were specified as random factors. A full random structure was specified for participants and items (3). If this model did not converge or singular fit, we trimmed it until it converged, by first removing correlations between factors, then interactions first for items then participants, then slopes (27; McGowan et al., 2022). We also calculated the conditional and marginal R^2^ values for each by using the MuMIn package (4). The conditional and marginal R^2^ values represent the proportion of variability accounted for by the full model including both fixed and random effects, and by the fixed effects alone, respectively.

**Figure 3.**
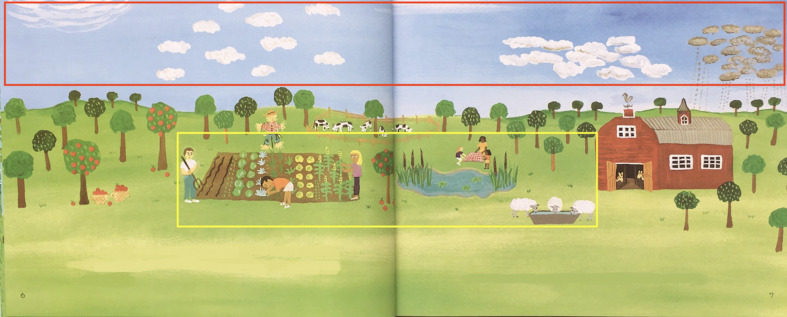
An example of the areas of interest. High-relevant area (red), which includes elements highly related to the theme, and low-relevant area (yellow), which includes elements less related to the theme.

For eye-movement measure analysis (32; 34), there were two areas of interest (AOI) (see Figure 3) which including high-relevant elements and low-relevant elements; four eye-movement measures were selected: percentage of total fixation duration (TFD%) , percentage of fixation count (FC%), visit count (VC) and average fixation duration (AFD). TFD% refers to the percentage between the TFD in a given AOI and the TFD on the whole page; FC% refers to the percentage between the sum of the FC in the AOI and the sum of the FC on the whole page; the VC refers to the total number of visits to the AOI; the AFD refers to the mean duration of all fixation points in the AOI.

## Results

### The effects of elements relevance on DPB’s comprehension

The children's vocabulary knowledge, as measured by the Peabody Picture Vocabulary Test, showed an average of 59.88 (*SE* = 0.70) and ranged from a maximum of 102 to a minimum of 30. For comprehension test, the results of descriptive statistics were reported in Table 1 and fixed effects estimations were reported in Table 2.

**Table 1.** Descriptive statistics of the comprehension test under the three conditions

Elements relevance	Mean	Standard Error
High-relevant animated	3.32	.22
Low-relevant animated	2.18	.25
Static	3.02	.25

There was a significant relevance effect of animated elements, such that animation of low-relevant elements led to lower comprehension compared to animation of high-relevant elements (*t*=3.75, *p*<.001) or static condition (*t*=2.67, *p*<.01), while the average score in the high-relevant animated and static conditions were comparable. There was also a significant effect of vocabulary such that children with higher vocabulary scored higher than children with lower vocabulary (*t*=2.16, *p* <.05). However, there were no significant interactions between any pair of elements relevance and vocabulary (*t*s<0.79, *p*s>.05), which shows that individual differences in vocabulary cannot modulate the relevance effect of animated elements on comprehension. Furthermore, the marginal and conditional R² values for the model were 0.11 and 0.46, respectively.

**Table 2.** Estimates of fixed effects for comprehension test as a function of conditions and vocabulary.

	*b*	*SE*	*t*	*CI*
Intercept	2.84	0.54	5.30*	[0.35, 2.23]
Comparison 1: High- vs. Low- relevant animated	1.06	0.28	3.75***	[-1.60, -0.51]
Comparison 2: Low-relevant animated vs. Static	-0.75	0.28	-2.67**	[-1.29, -.21]
Comparison 3: High-relevant animated vs. Static	0.30	0.28	1.08	[-0.24, 0.85]
Vocabulary	0.01	0.01	2.16*	[0.01, 0.02]
Comparison 1 × Vocabulary	0.01	0.02	0.39	[-0.03, 0.02]
Comparison 2 × Vocabulary	-0.01	0.02	-0.79	[-0.04, 0.02]
Comparison 3 × Vocabulary	0.01	0.02	0.40	[-0.03, 0.02]

Note. * *p* < 0.05, ** *p* < 0.01, *** *p* < 0.001

### The relationship between children’s eye movements and DPB’s comprehension

Linear regression analyses were conducted to separately examine the relationship between eye-movement behaviour within the high-relevant element AOI and comprehension test scores, as well as the relationship between eye-movement behaviour within the low-relevant element AOI and comprehension test scores. The results showed that both the TFD% and the FC% in the high-relevant element AOI had a positive predictive effect on the comprehension test (TFD%: *β* = 0.388, *t* = 3.708, *p*<0.001; FC%: *β* = 0.401, *t* = 3.982, *p*<0.001), while those in the low-relevant element AOI had a negative predictive effect (TFD%: *β* = -0.232, *t* =-2.216, *p=*0.029; FC%: *β* =-0.219, *t* = -2.174, *p* =0.032). Both the VC and the AFD in the low-relevant element AOI had a negative predictive effect on the comprehension test (VC: *β* = -0.329, *t* = -3.406, *p=*0.001; AFD: *β* = -0.298, *t* = -2.912, *p=*0.004), however that in the high-relevant element AOI had no significant predictive effect on the comprehension test (VC: *β* = -0.016, *t* = -0.163, *p=*0.871; AFD: *β* = 0.141, *t* = 1.375, *p=*0.172). In brief, the longer and more times the children fixated on the high-relevant elements, whereas the shorter and less times the children fixated on or visited the low-relevant elements, the higher the comprehension.

Furthermore, the Random Forest model was used to estimate the relative importance of these predictors and screen out the most sensitive and important eye movement measures on the comprehension test. Random forest is an algorithm based on classification tree (6) , and it can capture functional relations between dependent variables and predictors even in datasets with a small number of observations and a large number of predictors while avoiding two problems common for parametric regression approaches: overfitting and collinearity (29; 25).

**Figure 4.**
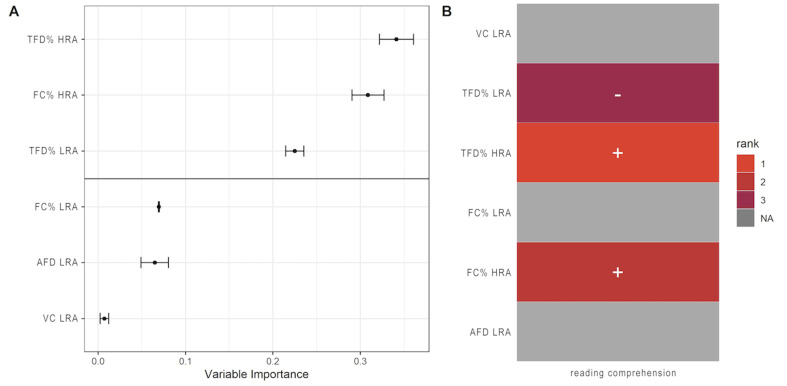
Relative variable importance obtained from Random Forests models for comprehension. (A) The variable importance scores are plotted in ascending order to show the rotated “Scree plot,” with the solid black horizontal line indicating the threshold chosen through visual inspection. Error bars represent the SE of variable importance scores obtained from multiple runs of forests. (B) A map representation of the variable importance where only variables above the threshold are colored according to their rank. TFD% HRA refers to Percentage of Total fixation duration on High-Relevant Area, TC% HRA refers to Percentage of Fixation Count on High-Relevant Area, TFD% LRA refers to Percentage of Total fixation Duration on Low-Relevant Area, TC% LRA refers to Percentage of Fixation Count on Low-Relevant Area, AFD LRA refers to Average Fixation Duration on Low-Relevant Area, and VC LRA refers to Visit Count on Low-Relevant Area.

The result was reported in figure 4 using the following two steps (see 25). First, we determined a threshold for variable importance by visually inspecting the gap in the sorted list of relative importance scores (shown as a horizontal line in Figure 5A). Second, we generated an image where the color-coded reflects the ranked relative importance of variables (in Figure 5B). It showed that the top three predictors which were TFD% and FC% on high-relevant area and TFD% on low-relevant area were distinguishable from the rest and they had the relative greater impact on comprehension.

### The relevance effect of animated elements relevance on eye-movement behavior

According to the aforesaid results, we further analyzed the relevance effect on the eye-movement measures which were top-three important predictors on comprehension (see Table 3 & 4).

**Table 3.** Eye-movements of high- and low- relevant elements areas under the three conditions.

	TFD% HRA ^a^	FC%HRA ^b^	TFD% LRA ^C^
High-relevant animated	56.0(1.7)	51.5(1.6)	18.8(1.5)
Low-relevant animated	47.4(1.6)	43.4(1.5)	29.9(1.6)
Static	54.5(1.7)	50.2(1.6)	19.7(1.4)

Note. ^a^ Percentage of Total fixation duration on high-relevant area, ^b^ Percentage of Fixation count on high-relevant are, ^c^ Percentage of Total fixation duration on low-relevant area

The main effect of elements relevance was showed on both the TFD% and FC% in high-relevant area. Specifically, children fixated shorter or less times on high-relevant elements area in the low-relevant animated condition compared to the static condition (TFD%: *t* = -2.95, *CI* = [-0.25, -0.04]; FC%: *t* = -2.85, *CI* = [-0.25, -0.04]); while there was no significant difference between low-relevant animated and high-relevant animated conditions or between high-relevant animated and static conditions (*ts* < 0.68). Notable, there was a marginal significant interaction between low- and high- relevant animated and vocabulary on measure of TFD% (*t* = -1.76, *CI* = [-0.001, -0.007]), such that children with higher vocabulary looked less on area of relevant elements in low- compared to high- relevant animated condition. Additionally, there was neither a main effect of vocabulary nor any interaction (*ts* < 1.21).

The main effect of elements relevance also showed on the TFD% in low-relevant elements area. In detail, children made longer fixation in the low-relevant animated condition than in the high-relevant animated condition (*t* = 4.14, *CI* = [0.38, 1.08]), or the static condition (*t* = 5.14, *CI* = [0.39, 0.89]). In addition, there was a significant main effect of vocabulary on TFD% (*t* = -2.38, *CI* = [-0.008, -0.001]), due to more visual attention on the area of low-relevant elements for children with lower vocabulary. Nevertheless, there was no interaction (*ts* < 0.96).

**Table 4.** Estimates of fixed effects for eye movements measures as a function of conditions and vocabulary.

		TFD% HRA ^a^	FC%HRA ^b^	TFD% LRA ^C^
Intercept	*b*	0.88	0.97	-2.28
	*SE*	0.10	0.11	0.20
	*t/z*	8.39***	9.09***	-11.16***
Comparison 1: High- vs. Low- relevant animated	*b*	0.07	0.05	-0.73
	*SE*	0.16	0.16	0.18
	*t/z*	0.42	0.33	-4.14***
Comparison 2: Low-relevant animated vs. Static	*b*	-0.15	-0.15	0.64
	*SE*	0.05	0.05	0.13
	*t/z*	-2.95**	-2.85**	5.14***
Comparison 3: High-relevant animated vs. Static	*b*	0.08	0.10	-0.08
	*SE*	0.14	0.14	0.15
	*t/z*	0.55	0.68	-0.55
Vocabulary	*b*	0.001	0.002	-0.004
	*SE*	0.001	0.001	0.002
	*t/z*	1.16	1.14	-2.38*
Comparison 1× Vocabulary	*b*	-0.003	-0.003	0.002
	*SE*	0.002	0.002	0.003
	*t/z*	**-1.76~**	-1.59	0.54
Comparison 2× Vocabulary	*b*	0.002	0.002	-0.001
	*SE*	0.002	0.002	0.003
	*t/z*	1.27	1.06	-0.34
Comparison 3× Vocabulary	*b*	0.001	0.001	-0.003
	*SE*	0.002	0.002	0.003
	*t/z*	0.52	0.56	-0.85
*R*^2^ （m）		.01	.01	.07
*R* ^2^		.70	.69	.71

Note. ^a^ refers to the percentage of total fixation duration on high-relevant area, ^b^ refers to the percentage of fixation count on high-relevant are, ^c^ refers to the percentage of total fixation duration on low-relevant area.

## Discussion

The present study investigated the impact of the relevance of animated elements on preschoolers' comprehension while engaging with digital picture books. The findings revealed that the animation of low-relevant elements led to lower comprehension scores compared to high-relevant animations and static condition. Eye-movement analysis further indicated that children who fixated less on high-relevant elements or more on low-relevant elements had poorer comprehension. Specifically, children fixated significantly less on high-relevant elements in the low-relevant animation condition compared to the static condition, indicating that the animation of low-relevant elements reduces children’s attention to crucial information. Meanwhile, children showed significantly more fixations and higher frequency of visits to low-relevant elements in the low-relevant animation condition compared to both the high-relevant animation and static conditions. This suggests that low-relevant animations lead to increased visual attention on less relevant elements, disrupting the construction of a mental representation of the narrative and interfering with the integration of auditory and visual information, ultimately resulting in a negative impact on comprehension. In conclusion, these results suggest that low-relevant animations in DPB can disrupt children’s comprehension by drawing their attention more to less relevant elements and reducing their focus on the crucial, high-relevant elements.

However, although we found that children’s comprehension in the high-relevant animation condition was significantly better than that in the low-relevant animation condition, there was no significant facilitating effect of the animation of high-relevant elements compared with the static condition (only existed a numerical trend on the test score: high-relevant is 3.32 and static is 3.03). This is inconsistent with the findings of Takacs and Bus (40), who found that the animation of relevant elements attract children’s attention and thus promote story comprehension. Our results based on eye-movement behavior showed that the animation of high-relevant elements did not significantly increase the children’s fixation on high-relevant elements nor reduce the fixation on the less-relevant elements, which is in line with the findings of Li et al. (2015), who suggested that repetitive visual stimuli may lose their attention-capturing effect over time. One possibility is that the animated attraction might gradually decreased with the repeated occurrence of similar elements across pages. In our experimental materials, each book revolved around a single theme, so the animated processing of high-relevant elements were mainly focused on one object. For example, most of the high-relevant elements in “*Clouds*” were clouds. With the repeated presentation of animated clouds in each page, their continuous attraction to children would be reduced, which may result in less sensitivity to the animations of related elements. On the contrary, the variety of less relevant elements was more diverse, such as people, animals, flowers and plants in the same books. Because of constant change, low-relevant elements can keep attracting the attention of children. This is to say; the effect of the animated elements may also be affected by the repeated occurrence of the elements per se. However, this explanation needs to be investigated directly in future studies. Besides, the findings are only based on data obtained from children aged 4 to 5 years old; therefore, further investigation is also necessary to determine if these effects vary in younger or older age group.

With regard to the individual differences, we found that the children with lower vocabulary knowledge had worse story comprehension; but for all children regardless of their vocabulary knowledge, their comprehension was interfered by animation of low-relevant elements, which is inconsistent with our expectation. However, results of eye movements showed a trend of interaction between individual difference and the relevance of animated elements, such that for children with lower vocabulary knowledge, their fixation on high-relevant elements decreased greater in low-relevant compared to high-relevant animated condition. It’s possible that children’s visual attention is more likely to be guided by irrelevant information, particularly those with lower vocabulary knowledge who may struggle more with comprehension. Furthermore, this also reflects the interaction between cognitive factors (e.g., prior knowledge) and sensory stimulation (e.g., animation or novelty) affecting children’s visual attention in the task of picture book reading. It is consistent with the current psychological idea that the dynamic interaction of Top-Down and Bottom-Up information controls where, how and to what we pay attention in the visual environment (14). Future research could also focus on the inhibitory control abilities of young children in relation to visual interference from low-relevant or irrelevant animated elements, as findings show that children with higher interference control skills exhibited superior memory performance, while distracting visual stimuli decreased performance, highlighting a close relationship between young children's working memory and visual attention (35).

To sum up, the results of this study support the hypothesis that relevance of animated elements can influence the DPB’s comprehension of preschoolers; and to some extent caused by directing their visual attention during the processing. We also found that the longer children look on the high-relevant elements, the better understand; on the contrast, the longer they look on the low-relevant elements, the poorer comprehend the story. What’s more, our data indicated that the positive effect of directing children’s attention to the high-relevant elements in an animated way is more important than that of guiding their attention away from the low-relevant elements. Therefore, it is necessary to take illustration’s relevance into consideration in DPB’ animated design and to guide children’s eye movement behaviour through purposeful design to make them pay more attention to the elements with high-relevance to the target knowledge. In addition, more diverse high-relevant elements could be designed to obtain children’s continuous and stable attention, as well as try to adopt an animated way more in line with how things change. These can not only bring children a good engaging experience, but also promote the acquisition of knowledge.

### Ethics and Conflict of Interest

The author(s) declare(s) that the contents of the article are in agreement with the ethics described in http://biblio.unibe.ch/portale/elibrary/BOP/jemr/ethics.html and that there is no conflict of interest regarding the publication of this paper.

### Acknowledgements

This research was supported by grant from the Philosophy and Social Science Foundation of Tianjin 2021 [52WJ210011].

We thank Yinghong Han for helping recruit participants, Guang Zhao for his helpful comments on this manuscript, and the anonymous reviewers for their valuable feedback and insightful suggestions, which greatly contributed to the improvement of this manuscript.
